# Supplemental Review of Practical Medicine; Selected and Arranged, with Commentaries

**Published:** 1820-12-01

**Authors:** 


### § IV. NERVOUS SYSTEM.

22. *Inordinate Irritability.** Every experienced surgeon must have met with instances where the inordinate sensibility of patients prevented them from undergoing necessary operations, even after they had been commenced. A mean of lessening this irritability is therefore a desideratum, and Mr. Wardrop proposes *venesectio ad deliquium animi* as the measure, founded on the following case.

"A young woman of robust form, had a tumour on the orbitar plate of the left frontal bone, the base adhering firmly to the bone, whilst the exterior portion was attached to the integuments, in which there was a small sinus leading into the interior of the tumour. The diseased mass did not exceed the bulk of an almond, but it was attended with great pain, and even cautiously touching the orifice of the sinus with a probe, excited violent irritation."

Several attempts were made to extirpate the tumour, but in vain; the moment the scalpel touched the integuments her struggles were invincible. Mr. Wardrop, a few days afterwards, had her bled in a warm room, from a large orifice. Fifty ounces of blood were abstracted before syncope ensued "which lasted a sufficient time to allow the tumour to be removed." "When the fainting went off, she would not believe that the operation had been performed, until she examined her face in a glass."

She remained pale and feeble for a few days, but "she rapidly recovered her wonted strength without in any way appearing to have suffered from the loss of blood."

* Some Observations on a mode of performing operations upon irritable patients, with a case, where the practice was successfully employed. By James Wardrop, Esq. Surgeon Extraordinary to the Prince Regent. *Medico-Chir. Transactions, Vol.* X.

While we admire Mr. Wardrop's resolution in having recourse to the foregoing measure, we should not be inclined to follow his example. A few years ago, we had an opportunity of seeing a fatal syncope induced, by abstracting a pretty large quantity of blood from a robust young man labouring under severe pulmonary inflammation in measles. From some inattention or mal-adroitness in the assistant, the patient was not laid horizontal immediately when syncope took place, and the consequence was, that the fainting ended in death. Independently, however, of the danger of such a termination of syncope, protracted to the extent of time required for even a very trifling operation, we apprehend that such a temporary quiescence of the circulation would not be very unlikely to produce coagulations of the fibrin of the blood, that might ultimately embarrass the function of the heart. We throw out these hints merely as cautionary ones; indeed, Mr. Wardrop himself states, that "it has not been his intention to recommend this mode of practice for general adoption." We cannot, however, entirely agree with our author, "that were circumstances equally urgent to suggest its employment, it may be ventured upon *fearlessly*."

### § V. VASCULAR SYSTEM.

23. *General Inflammation of the Arteries.** The Sieur Bach, of sanguineous temperament and tall stature, entered into the military service at the age of twenty, having previously enjoyed excellent health. During the next five or six years, he was exposed to great fatigue and all the vicissitudes of the seasons. Having retired from the service at the age of 27, he was seized the succeeding autumn, with acute rheumatism in the joints of the lower extremities, which was cured by repeated leeching and emollient cataplasms. Seven months afterwards, viz. in the month of March, 1816, Bach experienced pain in the epigastrium and between the shoulders, accompanied by a dry and frequent cough. His physicians fearing phthisis, which was hereditary in the family, put him on a strict regimen, which signally mitigated the symptoms abovementioned. In the month of July, however, they returned in an aggravated degree, accompanied now by palpitations of the heart. All these were again mitigated by

* *Revue Medicale.*

a strict regimen. In March, 1817, a new exacerbation was experienced. The epigastric pain was very acute, and extended to the loins; the action of the heart was violent, and rendered more so by the least exercise—cough very frequent—irregular chills and flushes of heat—feeling of constriction in the epigastric region. At the end of a few days an intense head-ache, with the development of fever, of the continued kind—pulse strong, full, and frequent—pulsation of the superficial arteries evident to the view—skin not hot, although the patient experienced a burning heat internally—countenance pale—appetite still preserved, especially for cold victuals and acidulous fruits. Dr. Barde, of Toulouse, now saw the patient, and ordered twelve leeches to the anus, with mucilaginous ptisans and spare diet. This regimen, with reiterated applications of leeches to the fundament, gradually mitigated the symptoms—the patient began to take a little exercise—and in a fortnight, he was able to return to his usual avocations. Still, however, the least fatigue produced oppression, and excited palpitations of the heart, which indeed never entirely subsided. The patient kept himself slightly bent forward, and frequently pressed his hand on the epigastrium, which, he said, rendered the pain more supportable. Stimulating rubefacients to the anterior and posterior parts of the chest gave more freedom to the respiration; and a blister to the left arm, kept open a long time, seemed to reduce a little the action of the heart.

In this state Bach continued till the summer of 1818, when the disorder took on a more alarming character. The oppression at the chest became very great—the cough constant and aggravated by fits—the patient could not lie in bed but in a half bent posture—the pulsations of the carotid, temporal, and radial arteries, were visible at several paces distance; while the action of the heart extended to the epigastrium, which it forcibly elevated at each pulsation.

The frequent application of leeches to the fundament and epigastrium afforded temporary, and only temporary relief. It was remarkable that, even in the height of the febrile paroxysms, the patient appeared pale. Emaciation now made rapid strides. In the night of the 24th October, the patient was seized with a violent chill, succeeded by intense heat, and danger of suffocation. Our author was summoned to him at 5 o'clock in the morning, and bled him immediately from the arm to a considerable extent, which produced a sudden calm. The blood was quickly covered with a thick crust. Two other venesections were practised within a fortnight, which was passed in a state of comparative tranquillity. But on the 18th day, he experienced an attack similar to the preceding, and was relieved by the same kind of depletion. These attacks, however, were renewed, from time to time, and in the beginning of the year 1819, his state became truly alarming. Drs. Viguerie and Cabirau were now called into consultation, and the opinion was, that the patient laboured under general inflammation of the arteries. Dropsy now supervened and the patient was unable to lie down; while syncope was induced by the least motion. He expired on the 6th May, 1819.

*Dissection.* The cavities of the pleura, pericardium, and peritoneum, contained a considerable quantity of water. The heart was enlarged, and its cavities morbidly dilated. The membrane lining the auricles and ventricles, was of a brownish colour, covered with a mucous substance, and streaked with deep red patches. On removing the mucosities, the membrane underneath was found detached at those points where the red patches were situated. In all the rest of its extent the membrane was observed to be thickened, and so tenaciously adherent to the muscular structure of the organ, that it was impossible to detach it thence. The valves, particularly those of the right ventricle, were infiltrated with a reddish mucosity. The inner membrane of the aorta, carotids and branches, subclavians, brachials and branches, was found thickened, indurated, of a deep red colour, and covered with a whitish purulent matter. Between the internal and muscular coats of the arteries was found a layer of yellow serosity of some consistence. The pulmonary arteries and veins presented similar appearances. The abdominal aorta, also, and all its branches, even to the lower extremities, exhibited the same phenomena on their inner membrane, and between that and the fibrous tunic. The trunks of the two cavæ, from the subclavian veins above, and the abdomen below, participated, in a very high degree, the inflammatory condition of the arteries. Their inner membrane and valves were thickened, red, and easily lacerable. The mucous membrane of the stomach and small intestines was of a reddish colour, and the blood-vessels strongly injected. There were also some marks of inflammation in several other portions of the abdominal viscera.

We have translated this case verbatim, because it is one of the best detailed histories on record of this curious and dangerous affection. Dr. J. P. Frank appears to have been the first who attributed to inflammation of the arteries a peculiar species of inflammatory fever. M. Portal next reported a case of inflammation of the inner tunic of the aorta, in a subject, where the eruption of measles had been suddenly repelled, and death ensued in a few days preceded by violent sense of suffocation and strong palpitations of the heart. In July 1819, M. Dolbant presented to the faculty of Medicine of Paris a thesis on arteritis, in which are detailed five instances of inflammation, more or less extensive, of the internal tunics of arteries. Our own countryman, however, Mr. Hodgson had previously related a few cases of a similar nature. M. Vaidy has reported an interesting case of this kind in the Journal Complimentaire for August 1819. None of those histories, however, are nearly so complete as that of Dr. Barde's which we have here detailed.

Inflammation of the arteries is sometimes developed after the sufferings occasioned by a great surgical operation. The three first cases related by M. Dolbant were of this kind. At other times, however, it takes place without any evident cause, as in the fourth and fifth cases of the same author, and those of M. Vaidy and Barde.

We may remark in conclusion that vascular depletion, in the case here detailed, appears to have been greatly neglected in the beginning; although the symptoms were signally mitigated by it when ultimately put in execution. It is evident that in such dangerous cases the most rigorous antiphlogistic measures can alone offer any chance of saving the patient's life. *Revue Medicale, No.* III. *Mai* 1820.

24. *Intermittent Fever.* The difficulty which is often experienced in retaining cinchona on the stomach, in fevers of type, renders it desirable to have substitutes for that medicine. We, ourselves, always employ the arsenical solution in such cases, but many practitioners have an unreasonable dread of this potent substance. Recently two substitutes for cinchona have been proposed—bitter almonds and coffee. Dr. Mylius* employed the former in 27 cases of quotidian and quartan fevers. Two were cured after the second dose—four after the third—nine after the fourth—four after the fifth—four after the sixth—two after the seventh—one after the eleventh—and one after the twelfth. An emetic was generally premised, and the dose of the remedy was ℥iss. or ℥ij. of the bitter almond formed into an eight ounce emulsion, and taken one hour before the access of the paroxysm. No relapse occurred.

Dr. Thomson† relates some cases of intermittent fever cured

---

* Bulletins of the Faculty of Medicine, No. IV.
† Ed. Journal, 62.

by coffee, in the island of Jamaica. The form recommended is an ounce of dried ground unroasted coffee, infused in a pint of water, and boiled down to four ounces. One ounce to be taken cold three times a day in the intervals of the fever.

Quassia was employed successfully by the same gentleman. The infusion is the most agreeable form; but Dr. T. has several times given it, finely rasped, to the extent of several drachms in the day, and never found it to irritate the stomach. It may be continued during the paroxysms of the fever, which is an advantage over cinchona.

25. *Cutaneous Diseases.** We are afraid that there is too much truth in the assertion of Dr. M. that, notwithstanding the classification and graphic delineations of cutaneous diseases, "little has been added to our stock of knowledge regarding their cure." The following is the mode of treatment lately pursued with more than ordinary success by our author.

"The principle upon which I have acted is, that cutaneous eruptions are composed of an infinite number of minute *ulcers*, which, of course, fall to be treated as such, according to the ordinary rules of surgery. From this view it will follow, that all siccant remedies, in whatever shape, are inadmissible, and that the eruptions must be kept open and clean, till the cure is completed. This purpose has been effected by friction, and by, as far as possible, secluding atmospheric air. The means I have adopted are these: I dip a sponge in luke-warm water, and after squeezing it hard, so that only dampness remains, I cover it with oatmeal. With this the parts are rubbed for some length of time, the sponge being frequently dipt in the oatmeal, and this operation is repeated two or three times a day, according to the urgency of itching or other symptoms. After being sufficiently rubbed, the parts are washed and gently dried. Oil is then applied, by means of a varnish-brush, and the parts covered up with slips of linen. I give the preference to oil made from cow-heel, on account of its tenacity, and because it can be constantly and easily procured in a fresh state. During the friction, should a little blood ooze out, the appearance need create no uneasiness, and the consequences are rather favourable than otherwise." 526.

When Dr. Morrison suggests that this treatment should be applied to *variolous* eruptions, in order to prevent the secondary fever, he betrays more of the hobby-horse, than of sound pathology. It may have been useful in what is de-

---

* Thomas Morison, M. D. *Ed. Journ.* 65.

☞ This paper was placed by mistake in this Section.

nominated psoriasis inveterata, especially when combined, as it here was, with blue pill and ex. colocynth. internally, together with saline aperients.

26. *Obliterated Aorta.** We believe that this case makes the third on record, of obstructed aorta, compatible with life. They go far to establish the general sufficiency of collateral circulation, for the purposes of the animal economy. Facts of this kind serve to increase our confideuce in the resources of nature, as well as extend the boundaries of art.

Dr. Goodisson, upon endeavouring, at the HOSPICE DE LA PITIE, to trace the origin of the inferior mesenteric artery, on the body of a female, discovered a hard tumour placed directly upon the line of the aorta, and which was ascertained to be a diseased state of this vessel. The aorta itself, from the origin of the inferior mesenteric artery downwards, together with the greater part of the iliacs, was obliterated. The vessel lay close, and was firmly attached to the spine, flattened posteriorly, but still preserving its convex form in front. There was a large quantity of gelatino-cartilaginous matter surrounding that part of the aorta and vena cava, together with the portions of the iliac arteries and veins, which were included in the disease. Some vegetations were observed on the mitral and tricusped valves of the heart; but no other material derangement. The aorta, at its arch, was expanded to nearly double its natural size. Internally it was studded with "gross patches of bone," and depositions of ossific matter were seen in different places along the course of the artery. A bony sheath encased the diseased portion of artery for about two inches, and was filled with a firm fleshy substance, resembling heart. The lumbar arteries given off above the obliteration were considerably increased in size, as were the intercostals, forming free inosculations with the mammary, which was also enlarged in calibre. The spermatic arteries were immensely increased.

*New Circuit of the Blood.* "The mammary arteries were a good deal enlarged, and, like the spermatic arteries, their course was beautifully marked by the serpentine convolutions which they formed. That of the left side was joined by a considerable branch from one of the intercostals at the superior anterior spinous process of the ilium. This branch took its usual course from the aorta, passing immediately

* A case of obliterated aorta. By. THOMAS GOODISSON, M. D. with some additional observations, by Philip Crampton, M. D. Surgeon General, &c. *Dublin Hospital Reports*, *Vol.* II.

along the external edge of the psoas parvus one half of its length, then passing between the transversalis and obliquus descendens, and continuing its course between those two muscles, till, arriving at the before-mentioned point, it joined the mammary, (or epigastric,) together with a branch of considerable size, passing from between the fourth or fifth lumbar vertebræ and another smaller one, which, passing across at right angles, the whole were conveyed together by the medium of the circumflexa ilii to the usual origin of this vessel in the external iliac." 198.

The general appearance of the body could not be said to indicate disease, and the viscera were all sound, with the exception of an abscess in the lungs, which had burst into the trachea and destroyed life. The lower extremities were full proportioned, and well supplied with blood. No history of the woman's complaint could be traced.

27. *Melancholia cured by accidental mercurial Salivation.** Dr. Burrows relates an interesting case of melancholia in a woman, 40 years of age, who, without any apparent moral causes, became unhappy, fanciful, and nervous, being dyspeptic in consequence of a sedentary life. She became persuaded at length that she had hernia. This was the predominant hallucination, and ended in an attempt at suicide, by precipitating herself from a window at a great height. The contusion sphacelated, and the discharge was profuse. During convalescence from the injury, and while the mental hallucination continued, an accidental mercurial ptyalism took place, from some calomel and squills exhibited as a diuretic: "Concurrent with this ptyalism was an amelioration of the mental disorder. She grew more cheerful, and every aberration by degrees vanished." She married afterwards, "but neither the joys, nor the hopes, nor the pains, nor the disappointments of the marriage state, have deranged the equanimity of her intellectual faculties."

Dr. Burrows saw a similar result in two or three other instances of insanity; but wisely cautions us against drawing any precipitate conclusions from these cases, though they may afford us a valuable hint on some occasions.

"The physical phenomena exhibited in insanity indicate, generally, a marked derangement of the vascular as well as of the nervous system. There is the plainest evidence, sometimes of increased, sometimes of decreased arterial action; and when the equilibrium of

* Dr. G. M. Burrows. *Med. Repos. Oct.* 1820.

the circulation is disturbed, the sensorium sympathizes, and the functions of the intellectual organs are often implicated.

"Mercury, by its acknowledged power of equalizing the circulation, may restore the balance between the vascular and nervous systems, and thereby remove that morbid condition of the brain, which, perhaps, originates intellectual derangement. The theory of the operation of mercury on the system, therefore, favours the presumption, that salivation induced by it, may terminate insanity, while no such expectation could be formed from spontaneous salivation.—*Med. Repos. p.* 278.

Dr. Burrows has repeatedly given mercury as an alterative, in insanity, where the functions of the digestive organs were faulty, and he thinks with advantage; but always in combination with other pharmaceutical remedies. He has since tried it constitutionally.

"But, including the three cases referred to in this paper, sanity has followed four times only in nine cases where mercurial salivation has been accomplished. My experience, therefore, of artificial salivation in intellectual disorders is still much too limited to speak confidently of its success. Yet enough perhaps is ascertained to encourage a more extended trial of it; and this I shall not omit when opportunity offers." 279.

These observations are valuable, as any from Dr. Burrows must always be.

### § VII. MEDICINAL AGENTS.

28. *Poisoning by Oxymurias Hydrargyri.** A lady, recovering from parturition, swallowed, by mistake, a lotion containing thirty grains of corrosive sublimate, and quickly felt a burning sensation in the stomach. She immediately irritated her throat with the finger, and threw up some fluid. Dr. Thomas was with her in a minute or two: but having a recollection that liver of sulphur was considered the best antidote for the poison in question, he posted off himself for a surgeon, and *three quarters of an hour* elapsed before they returned to the patient! An emetic of ipecacuan was now administered, and its action promoted by warm water, &c. In short, the woman recovered, but evidently more by the powers of Nature, than by the assistance of art. We are quite amazed how a physician could think of leaving a poisoned patient for three quarters of an hour to search for liver

* By Charles Thomas, M. D. *Ed. Journ. No* 65.

of sulphur, without flying to other antidotes nearer at hand, as eggs, milk, gum water, sugar and water, or even water itself, if none of the above were within reach. Surely this case is a sufficient commentary on what we last quarter said respecting the propriety of having a *toxicological chart* hung up in every medical man's house. No man can answer for his presence of mind on these occasions; nor should he trust to memory alone. Let him have the *litera scripta* ready to refer to, and then if he fails, he will have the consolation of having done what was right without delay.

29. *Mercurial Ointment.** Dr. Dean and some other American practitioners have lately been a good deal in the habit of employing the strong mercurial ointment, as an external application in severe cases of erysipelas. When inflammatory symptoms are urgent, they, of course, use depletory measures, and in all cases attend to the functions of the chylopoetic viscera, viewing the disease as, for the most part, dependent on derangement of the digestive organs. It is only as a useful auxiliary measure that the ointment abovementioned is employed. "It is to be freely rubbed into the parts occupied by the erysipelatous inflammation, and the limbs afterwards enveloped in fine linen cloths, upon which some of the ointment has been previously spread. This process is to be repeated twice or thrice a day." A few hours after the first application of the ointment the patient expresses much relief from the burning pain, heat, and itching. Upon a farther continuance of the remedy, the eruption usually ceases to spread, and assumes a more favourable aspect. The inflammation soon after subsides, and with it the general irritation of the system. Erysipelas is a disease very common in America, and therefore the above suggestion of our transatlantic brethren deserves a trial in this country.†

30. *Infusum Digitalis.*‡ "In acute diseases generally," says a zealous brother labourer in the field of medicine, "my trials of it do not offer many temptations to extend its

---

* Dr. A.T. Dean, *American Medical Recorder, July* 1820.

† We understand that in some fatal cases of erysipelas which occurred lately in St. George's Hospital, the internal coats of the blood-vessels were found inflamed.

‡ A practical physician.

employment; though in chronic affections, it often accomplishes all that can reasonably be expected from medicine." In reading the case to which these remarks are subjoined, I was struck with the patient's expression at the time he was taking an ounce of the infusion every fourth hour. "Take care!" said he, frequently, in a hurried yet cautious tone. He expired on the same day.

I should not have been struck with this expression, had it not happened, that I lost two patients about three years ago during the exhibition of this medicine: and although I was prescribing it within authorized limits, I mean the limits set to its exhibition by posological writers, my patients died under such suspicious circumstances, that I wrote the word "Cave!" under the pharmaceutical or posological mention of Digitalis, in that form, in every work in my possession. In one of the cases I had ordered only half an ounce every six hours; it was taken regularly during three days;—in the other, an ounce every eight hours, during two days; both cases were hydrothorax. No specific action was manifested on the heart, the stomach, the bowels, or kidneys, in either case, during the exhibition of the infusion; but both patients died suddenly in getting out of bed.

I cannot help considering the formula of the college too strong, although copied from Dr. Withering; by whom Digitalis was prescribed with an efficacy that entitles his authority to our special deference. But to do justice to that physician we shall keep in mind that he says, "one ounce of this infusion given twice a day is a medium dose." One and all of us, I suspect, when hard pressed by increasing disease, have incautiously exceeded in frequency the exhibition of this form of Digitalis, at least once in our lives. In a letter from Dr. Withering to Dr. Woodville, complaining of Dr. Letsom's incautious use of it, in the eight fatal cases recorded by Dr. L. in the Medical Memoirs, he says, "I am fully satisfied that had I prescribed it, as he has done, the effects in my hands, would have been equally useless and deleterious."

These remarks are calculated to induce caution, without destroying confidence, in this overpowering remedy, so highly extolled by all writers of the present day on hydrothorax.

A Practical Physician.

We have long been in the habit of employing the infusum digitalis, especially in dropsical affections, but we rarely exceed half a fluid ounce every eight hours. Dr. M'Lean seldom exhibited more than two fluid ounces in the day, and few physicians had more experience than he. *Rev.*

